# Identification of subgroups of patients with tension type headache with higher widespread pressure pain hyperalgesia

**DOI:** 10.1186/s10194-017-0751-0

**Published:** 2017-04-11

**Authors:** César Fernández-de-las-Peñas, Elena Benito-González, María Palacios-Ceña, Kelun Wang, Matteo Castaldo, Lars Arendt-Nielsen

**Affiliations:** 1 Department of Physical Therapy, Occupational Therapy, Physical Medicine and Rehabilitation, Universidad Rey Juan Carlos, Alcorcón, Spain; 2 Center for Sensory-Motor Interaction (SMI), Department of Health Science and Technology, Faculty of Medicine, Aalborg University, Aalborg, Denmark; 3 Department of Physical Therapy, University of Siena, Siena, Italy; 4 Poliambulatorio Fisiocenter, Collecchio, Parma Italy; 5 Facultad de Ciencias de la Salud, Universidad Rey Juan Carlos, Avenida de Atenas s/n, 28922 Alcorcón, Madrid Spain

**Keywords:** Tension type headache, Pain, Groups, Sensitization

## Abstract

**Background:**

Identification of subgroups of patients with different levels of sensitization and clinical features can help to identify groups at risk and the development of better therapeutic strategies. The aim of this study was to identify subgroups of patients with tension type headache (TTH) with different levels of sensitization, clinical pain features, and psychological outcomes.

**Methods:**

A total of 197 individuals with TTH participated. Headache intensity, frequency, and duration and medication intake were collected with a 4-weeks diary. Pressure pain thresholds were assessed bilaterally over the temporalis muscle, C5-C6 joint, second metacarpal and tibialis anterior muscle to determine widespread pressure pain hyperalgesia. The Hospital Anxiety and Depression Scale assessed anxiety and depression. The State-Trait Anxiety Inventory evaluated the state and trait levels of anxiety. The Headache Disability Inventory evaluated the burden of headache. Health-related quality of life was determined with the SF-36 questionnaire. Groups were considered as positive (three or more criteria) or negative (less than three criteria) on a clinical prediction rule: headache duration <8.5 h/day; headache frequency <5.5 days/week; bodily pain <47 and vitality <47.5.

**Results:**

The ANCOVA revealed that subjects in group 1 (positive rule, *n* = 89) exhibited longer headache history, shorter headache duration, lower headache frequency, higher widespread pressure hyperalgesia, higher anxiety trait levels, and lower quality of life (all, *P* < 0.01) than those subjects within group 2 (negative rule, *n* = 108). Differences were similar between men and women.

**Conclusions:**

This study identified a subgroup of patients with TTH with higher sensitization, higher chronicity of headaches and worse quality of life but lower frequency and duration of headache episodes. This subgroup of individuals with TTH may need particular attention and specific therapeutic programs for avoiding potential chronification.

## Background

Tension-type headache (TTH) affects up to 80% of the general population at sometime during their life and shows a global prevalence in adults of 42% [1]. The general health costs for headaches in Europe were €13.8 billion, most related to migraine and TTH [2]. In fact, TTH has been found to be the second most prevalent disorder in the world [3]; although it has received far less attention than migraine.

Although the mechanisms underlying the transition from episodic to chronic TTH are not fully understood, the existing literature supports that sensitization mechanisms play an important role [4, 5]. In fact, the most accepted theory is that frequent episodic TTH is peripheral dominant whereas chronic TTH involves more central components [6]. This hypothesis is based on several studies supporting the presence of pressure pain hyperalgesia in the trigemino-cervical area as a manifestation of altered nociceptive pain processing in TTH [7]. Nevertheless, pressure pain hypersensitivity within the trigeminal area may reflect both peripheral and central sensitization processes. The presence of widespread pressure pain hypersensitivity in distant pain-free areas in TTH supports the role of central sensitization [8, 9].

Identification of subgroups of patients with different levels of sensitization can help to better understanding of chronic and complex pain conditions, such as TTH, and to identify better therapeutic strategies. For instance, a subgroup of individuals with knee osteoarthritis (those with higher pain intensity but minimal radiographic change in the knee) exhibiting strong sensitization has been identified [10]. In individuals with chronic whiplash associated disorders higher widespread pressure sensitivity and cold sensitivity have been associated with higher disability levels [11]. There is a lack of studies investigating sub-classification in individuals with TTH. A clinical prediction rule to identify women with TTH who will be likely to respond favorably to a particular manual therapy approach was identified, but not validated [12]. The results identified 4 variables that were predictive of a positive outcome: headache duration <8.5 h/day, headache frequency < 5.5 days/week, bodily pain <47 points and vitality <47.5 points. If a patient presented with 3 of these 4 variables, the positive likelihood ratio was 3.4 (95%CI 1.4, 8.0) with a posttest probability of success of 80% [12]. It is possible that these variables can be also able to identify groups of patients with TTH with different levels of sensitization or clinical features. In fact, a similar procedure was recently observed in women with carpal tunnel syndrome where those positive on a clinical prediction rule for physical therapy exhibited higher pressure and thermal pain hyperalgesia and higher depressive levels than those negative on the rule [13]. Therefore, the aim of the current study was to characterize groups of patients with TTH by determining if those variables previously associated with a positive outcome for manual therapy are also able to identify patients with TTH with more central sensitization and worse clinical features.

## Methods

### Participants

A cross-sectional study was conducted. Individuals with headache were recruited from different university-based hospitals between January 2015 and December 2016. All diagnoses were performed following the criteria of the International Classification of Headache Disorders (ICHD-III beta, 2013) by neurologists, expert in headaches [14]. To be included subjects had to exhibit all pain features of TTH; report no more than one among photophobia, phonophobia or mild nausea, and no moderate/severe nausea or vomiting as requested by the ICHD-III diagnostic criteria [14]. A 4-weeks headache diary was used to substantiate the diagnosis and to obtain the headache features [15]. On the headache diary, participants registered the number of days with headache (days/week), the duration of headache episodes (hours/day), and the intensity of pain of each episode on an 11-points numerical pain rate scale (NPRS; 0: no pain; 10: the worst unimaginable pain) [16]. Further, the use of symptomatic and preventive medication intake was also recorded in the diary.

Participants were excluded if presented any of the following criteria: 1, any other primary or secondary headache including medication overuse headache as defined by the ICHD-III; 2, previous cervical or head trauma; 3, cervical herniated disk or cervical osteoarthritis on medical records; 4, systemic medical disease; 5, fibromyalgia syndrome; 6, had received physical therapy or anesthetic blocks in the head/neck within the previous 6 months; or, 7, pregnancy. All subjects read and signed a written consent form prior to their participation in the study. The study design was approved by the local Ethics Committees (URJC 23/2014, HRJ 07/14, Aalborg N20140063, CESU 5/2015) and was conducted following the Helsinki declaration.

Evaluations were conducted when patients were headache-free or, in those with a high frequency of headaches, when the intensity of pain was ≤3 points on the NPRS. They were asked to avoid any analgesic or muscle relaxant 24 h prior to examination. No change was made on their regular pharmacological treatment.

### Pressure pain thresholds

An electronic pressure algometer (Somedic® Algometer, Sollentuna, Sweden) was used to measure pressure pain thresholds (PPT) over the temporalis muscle, C5-C6 zygapophyseal joint, second metacarpal and tibialis anterior muscle. PPT is defined as the minimal amount of pressure where a sense of pressure first changes to pain. Participants were instructed to press the “stop-button” of the algometer as soon as the pressure resulted in a first sensation of pressure and pain. Pressure was increased at a rate of approximately 30 kPa/s. The mean of 3 trials on each point, with a 30 s resting period for avoiding temporal summation of pain [17], was calculated and used for the main analyses. The order of assessment was randomized between participants and the assessor was blinded to any other outcome. Participants practiced first on the wrist extensors of the right forearm. The reliability of pressure algometry has been found to be high [18, 19].

### Headache Disability Inventory (HDI)

The HDI assesses the burden of headache using 25 items that inquire about the impact of headache on emotional functioning and daily activities [20]. Possible answers for each item include YES (4 points), SOMETIMES (2 point) or NO (0 points). Thirteen items evaluate the emotional burden of the headache (HDI-E, maximum score: 52), and the remaining 12 items the physical burden (HDI-P, maximum score: 48). A greater score suggests a greater burden of the headache. The HDI has exhibited good stability at short and long-terms [21].

### State-Trait Anxiety Inventory (STAI)

The STAI is a 40-items scale assessing the state (items l-20, STAI-S) and trait (items 21–40, STAI-T) levels of anxiety [22, 23]. The STAI-S evaluates relatively enduring symptoms of anxiety where subjects use a 4-points response scale ranging from “not at all” to “very much”, to indicate the extent to which they experience each emotion. The STAI-T scale measures stable propensity to experience anxiety and tendencies to perceive stressful situations as threatening. It consists of 20 statements requiring individuals to rate how they generally feel on a 4-points scale. Higher scores indicate greater state and/or trait anxiety levels. Both scales have good internal consistency and excellent test-retest reliability [24].

### Health-related quality of life

The Short-Form Health Survey 36 (SF-36) was used to assess health-related quality of life. The SF-36 is a self-administered, 36-items questionnaire that measures health-related functions on eight domains: physical function, physical role, bodily pain, general health, vitality, social function, role-emotional, and mental health [25]. After summing Likert-scaled items, each domain is standardized ranging from 0 to 100 points according to established international guidelines where higher scores represent better quality of life [26].

### Sub-grouping

Patients were grouped according if they were positive or negative in the rule using identical criteria to those previously reported [12]: headache duration <8.5 h/day; headache frequency <5.5 days per week; bodily pain <47 points and vitality <47.5 points in the domains of the SF-36 questionnaire. Patients who met at least three of the four criteria were classified as positive on the rule (Group 1), whereas patients meeting two or fewer criteria were classified as negative on the rule (Group 2).

### Data analysis

Statistical analysis was performed using SPSS software version 20.0 (Chicago, IL, USA). Patients were grouped according to the rule [12]. Differences between groups in clinical features, burden of headache (HDI-E, HDI-P), depression (HADS-D), anxiety (HADS-A, STAI-T, STAI-S) and each domain of SF-36 questionnaire were compared using one-way analysis of covariance (ANCOVA) with gender as covariate, and *χ*2 test of independence for categorical data. A two-way ANCOVA was used to evaluate differences in PPTs with side (right/left) as within-subjects factor, group (positive or negative rule) as the between-subjects factor and gender as covariate. The normality and homogeneity criteria were checked for dependent variables with Kurtosis and Skewness for the normality and Levene’s test for the homogeneity criteria. Separate ANOVAs were performed for each variable. As multiple comparisons were conducted in the main analysis, a Bonferroni-corrected alpha level of 0.025 (2 independent-samples t tests) was required to support the validity of the sub-groups.

## Results

Two hundred and twenty-five (*n* = 225) subjects with headache were screened for possible eligibility criteria. Of these, 197 patients (72% women) satisfied all the eligibility criteria, agreed to participate and signed the written informed consent. The reasons for exclusion were co-morbid migraine (*n* = 15), fibromyalgia (*n* = 5), medication overuse headache (*n* = 4), or previous whiplash (*n* = 4). One hundred and nine (*n* = 109, 56.5%) were classified as frequent episodic tension type headache (FETTH), and 88 (44.5%) were classified as chronic tension type headache (CTTH) accordingly to the ICHD-III diagnostic criteria. Fifty-five (28%) were taking prophylactic intake, i.e., amitriptyline, on a regular basis, and 136 (69%) took symptomatic medication, i.e., NSAIDs, during the headache episodes. Eighty-nine individuals (45%) were classified as group 1 (positive rule) whereas 108 (55%) were classified as group 2 (negative rule).

### Headache clinical features and medication intake

Table 1 summarizes demographic and clinical data of each group in the total sample. The ANCOVA revealed significant differences between groups for years with headache (*F* = 5.748; *P* = 0.02), headache duration (*F* = 7.836; *P* < 0.001) and headache frequency (*F* = 17.148; *P* < 0.001), but not for age (*F* = 0.813; *P* = 0.488) or headache intensity (*F* = 0.904; *P* = 0.333): patients in group 1 showed longer history of headache, shorter headache duration, and lower frequency of episodes than those patients within group 2. No interaction of gender was observed for either outcome (age: *F* = 0.367, *P* = 0.545; years with headache: *F* = 0.573, *P* = 0.450; headache intensity: *F* = 0.919, *P* = 0.433; headache duration: *F* = 0.182, *P* = 0.670; headache frequency: *F* = 0.187, *P* = 0.666). A significant (*χ*2 = 11.594; *P* < 0.001) greater proportion of FETTH subjects were included in group 1.

**Table 1:** Clinical features, medication intake, psychological and related-disability outcomes in patients with tension-type headache by group

	Group 1 (Rule positive, *n* = 89)	Group 2 (Rule negative, *n* = 108)
Clinical Pain Features		
Gender (male/female) n (%)	22 (25%)/67 (75%)	33 (30%)/75 (70%)
Age (years)	44.8 (41.5, 48.1)	45.8 (42.8, 48.8)
Headache history (years)*	12.9 (10.0, 15.8)	8.8 (6.9, 10.8)
Headache intensity (0-10)	5.3 (4.6, 6.0)	5.1 (4.7, 5.5)
Headache frequency (days/)*	3.7 (3.2, 4.2)	6.0 (5.5, 6.5)
Headache duration (hours per attack)*	5.7 (5.0, 6.4)	8.1 (7.3, 8.9)
FETTH/CTTH n (%)*	60 (67.5%)/29 (32.5%)	49 (45.5%)/59 (55.5%)
Preventive medication (yes/no) n (%)	24 (27%)/65 (73%)	31 (28.5%)/77 (71.5%)
Symptomatic medication (yes/no) n (%)	61 (68.5%)/28 (32.5%)	75 (69.5%)/33 (31.5%)
Psychological and disability-related outcomes		
HADS-D (0–21)	7.8 (6.8, 8.7)	7.8 (6.8, 8.8)
HADS-A (0–21)	9.6 (8.7, 10.6)	10.0 (8.9, 11.1)
HDI-P (0–48)	24.9 (22.2, 27.6)	23.1 (20.5, 25.7)
HDI-E (0–52)	19.7 (16.6, 22.9)	18.3 (15.5, 21.1)
STAI-T (0–60)*	25.8 (23.8, 27.8)	22.1 (20.7, 23.5)
STAI-S (0–60)	21.3 (19.7, 22.9)	21.8 (20.6, 23.0)

*HADS* Hospital Anxiety and Depression Scale (*D* Depression; *A* Anxiety), *HDI* Headache Disability Inventory (*P* Physical; *E* Emotional), *STAI* State-Trait Anxiety Inventory (*T* Trait; *S* State)

Values are expressed as means (95% confidence interval); *Significant differences between groups (ANOVA, *P* < 0.01)

Finally, no significant differences in the distribution of gender (*χ*2 = 0.826; *P* = 0.363) or medication intake (preventive drug: *χ*2 = 0.441, *P* = 0.507; symptomatic drug: *χ*2 = 0.056, *P* = 0.813) were found between groups.

### Burden of headache and mood disorders

Patients in group 1 exhibited higher anxiety trait levels (STAI-T) than those within group 2 (*F* = 7.090; *P* = 0.009). No significant differences in the burden of headache (HDI-P: *F* = 1.240, *P* = 0.297; HDI-E: *F* = 0.499, *P* = 0.481), depression (HADS-D: *F* = 0.010, *P* = 0.982), or anxiety levels (HADS-A: *F* = 1.024, *P* = 0.384; STAI-S: *F* = 0.279, *P* = 0.599) were found (Table 1). Gender did not influence the results (HDI-E: *F* = 0.438, *P* = 0.509; HDI-P: *F* = 0.144, *P* = 0.705; HADS-D: *F* = 0.489, *P* = 0.485; HADS-A: *F* = 0.013, *P* = 0.909; STAI-S: *F* = 0.038, *P* = 0.847; STAI-T: *F* = 1.899, *P* = 0.171).

### Widespread pressure pain sensitivity

**Fig. 1:**
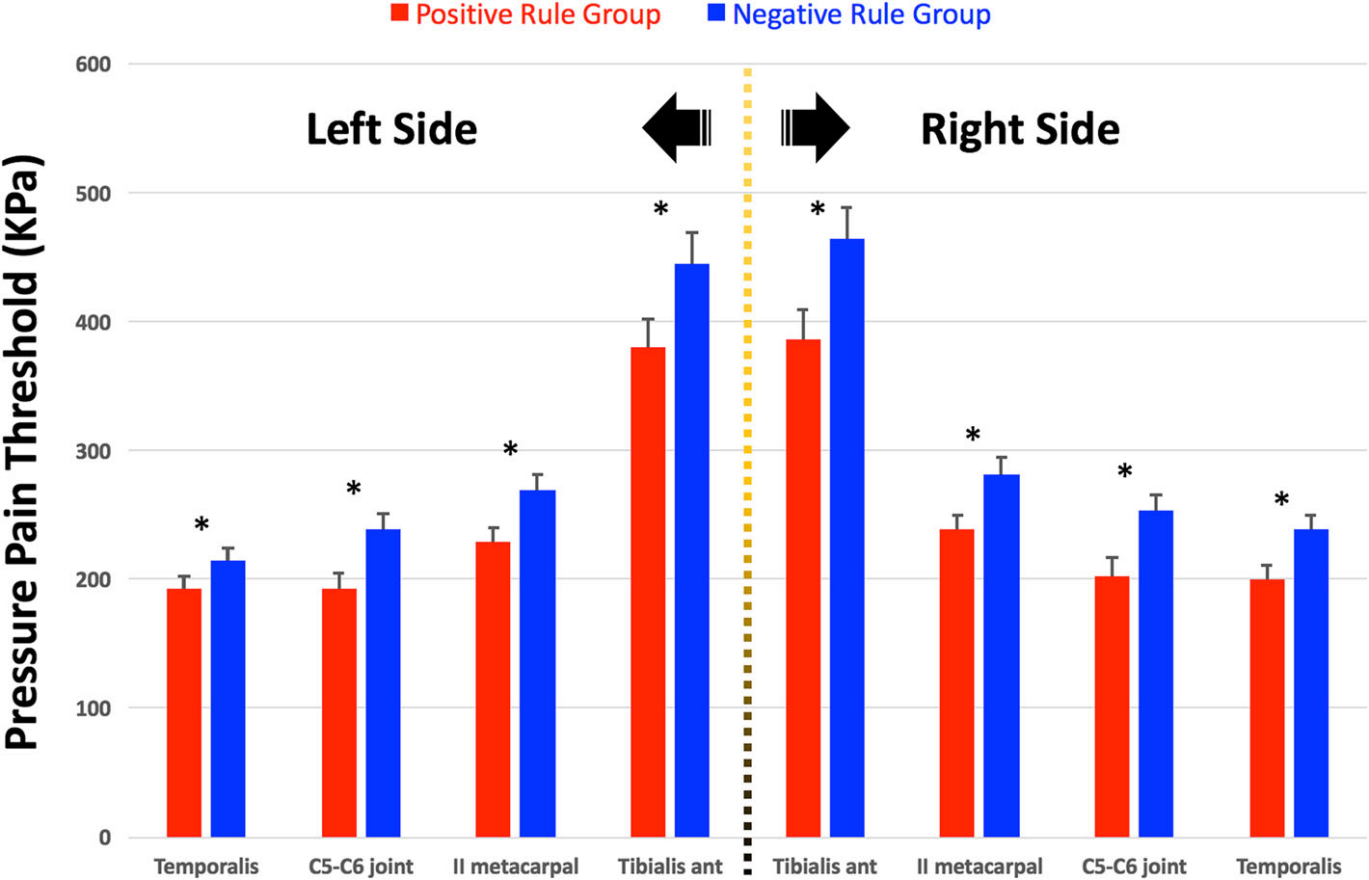
Differences in widespread pressure pain thresholds (kPa) between patients positive (group 1) or negative (group 2) in the rule. Data are expressed as means and standard error (SE). * Significant differences between groups (*P* < 0.01)

The two-way ANCOVA revealed significant differences between groups, but not between sides, for PPTs over all the points: temporalis muscle (group: *F* = 9.576, *P* = 0.002; side: *F* = 1.249, *P* = 0.304), C5-C6 joint (group: *F* = 12.739, *P* < 0.001; side: *F* = 0.819, *P* = 0.366), second metacarpal (group: *F* = 14.849, *P* < 0.001; side: *F* = 1.026, *P* = 0.312), tibialis anterior muscle (group: *F* = 10.626, *P* < 0.001; side: *F* = 0.346, *P* = 0.557). No significant side * group interactions were found. Patients within group 1 exhibited bilateral lower widespread PPT than those within group 2 (Fig. 1). A significant effect of gender was also found: temporalis muscle (*F* = 13.15, *P* < 0.001), C5-C6 joint (*F* = 12.46, *P* < 0.001), second metacarpal (*F* = 16.317, *P* < 0.001), tibialis anterior muscle (*F* = 11.435, *P* < 0.001). No group * gender interaction was either observed. PPTs were significantly lower in women than in men in both groups. Table 2 summarizes PPTs in all the assessed points within each group.

**Table 2:** Differences in pressure pain thresholds (PPT, kPa) between individuals with tension-type headache by group

	Temporalis muscle*	C5-C6 zygapophyseal joint*	Second metacarpal*	Tibialis anterior muscle*
Group 1 (Rule positive, *n* = 89)				
Right side	200.0 (179.6, 220.3)	220.4 (174.5, 230.2)	238.7 (216.6, 261.0)	386.0 (341.2, 430.9)
Left side	192.0 (171.6, 212.3)	192.4 (164.6, 220.2)	229.2 (207.1, 251.5)	379.7 (334.9, 424.6)
Group 2 (Rule negative, *n* = 108)				
Right side	238.7 (220.1, 257.4)	253.0 (227.6, 278.5)	281.8 (261.6, 302.0)	464.1 (423.0, 505.2)
Left side	214.5 (195.8, 233.1)	238.5 (213.0, 263.9)	269.4 (248.9, 289.8)	444.6 (403.1, 486.1)

Values are expressed as means (95% confidence interval)

*Significant differences between both groups (2-two way ANOVA test, *P* < 0.001)

### Health-related quality of life

The ANCOVA revealed significant differences in all domains of the SF-36 questionnaire (physical function: *F* = 6.600, *P* = 0.011; physical role: *F* = 4.428, *P* = 0.014; bodily pain: *F* = 7.654, *P* < 0.001; general health: *F* = 4.715, *P* = 0.018; vitality: *F* = 8.805, *P* < 0.001; social function: *F* = 8.076 *P* < 0.001; role-emotional: *F* = 7.099, *P* = 0.001) except mental health (*F* = 1.503, *P* = 0.195). Patients from group 1 exhibited lower quality of life than those patients from group 2 (Table 3). No effect of gender was observed for any SF-36 domain (physical function: *F* = 0.045, *P* = 0.833; physical role: *F* = 0.1704, *P* = 0.747; bodily pain: *F* = 0.108, *P* = 0.743; general health: *F* = 0.096, *P* = 1.482; vitality: *F* = 0.661, *P* = 0.417; social function: *F* = 0.890, *P* = 0.347; role-emotional: *F* = 0.652, *P* = 0.761) mental health: *F* = 0.659, *P* = 0.418).

**Table 3:** Differences in health-related quality of life in individuals with tension-type headache by group

	Group 1 (Rule positive, *n* = 89)	Group 2 (Rule negative, *n* = 108)
Physical Function (0–100)*	72.9 (67.6, 78.2)	83.7 (79.4, 87.8)
Physical Role (0–100)*	44.9 (36.7, 53.1)	58.7 (51.0, 66.4)
Bodily pain (0–100)*	39.4 (35.6, 43.2)	61.1 (57.0, 65.2)
General Health (0–100)*	52.9 (48.2, 57.6)	60.3 (56.3, 64.3)
Vitality (0–100)*	40.8 (37.1, 44.6)	59.1 (54.7, 63.5)
Social Function (0–100)*	58.0 (53.0, 63.0)	72.3 (68.5, 78.0)
Role Emotional (0–100)*	52.3 (43.4, 61.2)	51.1 (63.8, 78.3)
Mental Health (0–100)	55.4 (50.9, 59.9)	59.7 (55.5, 64.0)

Values are expressed as means (95% confidence interval)

*Significant differences between groups (ANOVA, *P* < 0.02)

## Discussion

The current study has identified a subgroup of patients with TTH with higher widespread pressure pain hyperalgesia, longer headache history and lower health-related quality of life but lower frequency and shorter duration of headache episodes. This classification was conducted accordingly to a clinical prediction rule originally developed for identifying women with TTH who were likely to respond favorably to a manual therapy targeting muscle tissues [12].

The group of patients with TTH exhibiting higher pressure hyperalgesia and worse quality of life was identified by applying a simple classification rule [12]. This classification system could potentially help to identify patients with TTH at a higher risk for developing more severe TTH (e.g., transition from episodic to chronic). In fact, it is also possible that this subgroup of patients with higher levels of sensitization would be more susceptible for developing widespread symptoms representing the 35–44% of sufferers with TTH presenting co-morbid fibromyalgia syndrome [27]. This hypothesis would be also supported by the worse health-related quality of life experienced by this subgroup of patients albeit they exhibited lower frequency and shorter duration of headache episodes since patients with TTH and co-morbid widespread symptoms experience worse health-related quality of life [28]. This maybe particularly relevant for the lower scores in one of the domains of the rule, bodily pain, which represents the experience of body pain symptoms by the patient. Nevertheless, the presence of higher sensitization in patients with lower frequency of headaches is contrary to what is observed in patients with migraine and co-morbid fibromyalgia syndrome since a higher frequency of migraine attacks enhances both hyperalgesia and widespread pain symptoms [29]. We do not currently know if the presence of widespread hypersensitivity appears before or after the increase of the frequency of headaches, or both processes are interconnected and therefore one promotes the other.

The presence of higher widespread pain sensitization may suggest different underlying mechanisms in this group of patients. It is accepted that prolonged nociception from peripheral tissues, i.e., muscles, is the main responsible for triggering centralized sensitization mechanisms and the evolution from the episodic to the chronic form [6, 30]. In the chronification process, the frequency of the headache episodes is found to play an important role [31]. However, our study found that the subgroup of patients exhibiting higher widespread pressure pain hypersensitivity reported lower frequency of headaches. This is supported by the fact that there was a greater distribution of patients with FETTH within group 1. Other possibility is that higher chronicity (history with pain symptoms) of headache can also explain higher sensitization levels [32]. We also observed that the group of patients with TTH with higher sensitization levels also exhibited a longer history of headache, supporting this hypothesis. This finding would suggest that, not only the frequency of headaches as previously reported, but also the chronicity of symptoms, may be also relevant for the development of central sensitization. Therefore, early therapeutic interventions for preventing development of central sensitization should be encouraged in this subgroup. An interesting finding was that the identified subgroup of patients with TTH with higher levels of sensitization also exhibited higher trait anxiety. Since stress is one of the most common trigger and aggravating factors of TTH [33, 34], it is possible that this subgroup of subjects would be more susceptible to stressful situations and therefore promoting development of central sensitization. Therefore, psychological approaches targeting anxiety trait levels should be also implemented in this subgroup of patients. Finally, it is also possible that this subgroup of patients with TTH exhibits different neurotransmitter concentrations or differences in brainstem processing [35] explaining the altered nociceptive processing; although this hypothesis should be investigated in future studies.

Although this is the first study investigating a classification system in patients with TTH, we should recognize some potential limitations. First, we recruited our patients from tertiary care hospitals; therefore, multi-center studies including individuals from the general population would help to extrapolate the results. Second, we only tested the response to pressure stimulation as it has been previously found that pressure pain sensitivity is a clear feature of TTH [6, 7]. It would be interesting to investigate other outcomes of central sensitization, e.g., thermal pain thresholds, conditioning pain modulation (CPM) or nociception flexor reflex (NFR) in these subgroups of patients with TTH.

## Conclusions

Patients with TTH who meet a clinical prediction for group 1 (at least 3 of the criteria) tended to exhibit higher widespread pressure hyperalgesia, longer headache history, higher trait anxiety levels, and worse quality of life but lower frequency and shorter duration of headache episodes than those within group 2 (met two or fewer criteria).

## Abbreviations

ANOVA: Analysis of variance
CI: Confidence interval
CTT: Chronic tension type headache
FETTH: Frequent episodic tension type headache
HADS: Hospital Anxiety and Depression Scale
HDI: Headache Disability Inventory
ICHD3: International Classification of Headache Disorders, third edition
NPRS: Numerical pain rate scale
PPT: Pressure pain thresholds
SF-36: Short-Form Health Survey 36
STAI: State-Trait Anxiety Inventory
TTH: Tension-type headache
